# Retraction: Gm5820, an antisense RNA of FGF1, suppresses FGF1 expression at the posttranscriptional level to inactivate the ERK/STAT3 pathway and alleviates neuropathic pain in mice

**DOI:** 10.1039/d1ra90034j

**Published:** 2021-01-26

**Authors:** Laura Fisher

**Affiliations:** Royal Society of Chemistry Thomas Graham House, Science Park, Milton Road Cambridge CB4 0WF UK advances-rsc@rsc.org

## Abstract

Retraction of ‘Gm5820, an antisense RNA of FGF1, suppresses FGF1 expression at the posttranscriptional level to inactivate the ERK/STAT3 pathway and alleviates neuropathic pain in mice’ by Xin Zhang *et al.*, *RSC Adv.*, 2019, **9**, 28364–28376, DOI: 10.1039/C9RA03791H.

The Royal Society of Chemistry hereby wholly retracts this *RSC Advances* article due to concerns with the reliability of the data. The images in the article were screened by an image integrity expert. The western blots in the paper are very regular-shaped and do not look genuine. Furthermore, the blots and many other features of the article were found to closely resemble the blots and features of other articles, which is highly unexpected given that there are completely different author lists for these articles.

The authors were asked to provide the raw data for this article, but did not respond. Given the significance of the concerns about the validity of the data, and the lack of raw data, the findings presented in this paper are not reliable.

The authors have been informed but have not responded to any correspondence regarding the retraction.

Signed: Laura Fisher, Executive Editor, *RSC Advances*

Date: 15^th^ January 2021

## Supplementary Material

